# “Alphabet” Selenoproteins: Their Characteristics and Physiological Roles

**DOI:** 10.3390/ijms242115992

**Published:** 2023-11-06

**Authors:** Carmen Beatrice Dogaru, Corina Muscurel, Carmen Duță, Irina Stoian

**Affiliations:** Department of Biochemistry, Carol Davila University of Medicine and Pharmacy, 050474 Bucharest, Romaniairina.stoian@umfcd.ro (I.S.)

**Keywords:** selenium, selenoproteins, oxidoreductase, oxidative stress

## Abstract

Selenium (Se) is a metalloid that is recognized as one of the vital trace elements in our body and plays multiple biological roles, largely mediated by proteins containing selenium—selenoproteins. Selenoproteins mainly have oxidoreductase functions but are also involved in many different molecular signaling pathways, physiological roles, and complex pathogenic processes (including, for example, teratogenesis, neurodegenerative, immuno-inflammatory, and obesity development). All of the selenoproteins contain one selenocysteine (Sec) residue, with only one notable exception, the selenoprotein P (SELENOP), which has 10 Sec residues. Although these mechanisms have been studied intensely and in detail, the characteristics and functions of many selenoproteins remain unknown. This review is dedicated to the recent data describing the identity and the functions of several selenoproteins that are less known than glutathione peroxidases (Gpxs), iodothyronine deiodinases (DIO), thioredoxin reductases (TRxRs), and methionine sulfoxide reductases (Msrs) and which are named after alphabetical letters (i.e., F, H, I, K, M, N, O, P, R, S, T, V, W). These “alphabet” selenoproteins are involved in a wide range of physiological and pathogenetic processes such as antioxidant defense, anti-inflammation, anti-apoptosis, regulation of immune response, regulation of oxidative stress, endoplasmic reticulum (ER) stress, immune and inflammatory response, and toxin antagonism. In selenium deficiency, the “alphabet” selenoproteins are affected hierarchically, both with respect to the particular selenoprotein and the tissue of expression, as the brain or endocrine glands are hardly affected by Se deficiency due to their equipment with LRP2 or LRP8.

## 1. Introduction

The biology and biochemical effects of selenium, as an essential micronutrient discovered by the Swedish scientist Jöns Jacob Berzelius in 1817, are largely accomplished by a class of proteins that contain selenium, named selenoproteins, involved in numerous different molecular pathways, biochemical processes, physiological functions, and pathological conditions. Selenoproteins are present in eukarya, archaea, bacteria, and even viruses. Most of these selenoproteins have oxidoreductase activity and contain one selenocysteine (Sec, U) residue, with one notable exception, i.e., selenoprotein P (SELENOP), which has 10 Sec residues.

In 1974, the American biochemist Thressa Campbell Stadtman became a pioneer of selenium biochemistry for her discovery of selenocysteine, which has thus become the twenty-first “naturally occurring” amino acid [1].

The discovery of selenocysteine was proof that, metaphorically speaking, “every end is a new beginning”, since the UGA stop codon, whose function is to terminate translation, is decoded as Sec, which is afterward inserted in selenoproteins, and so a new era of numerous capital research and discoveries about selenoproteins has begun.

**Figure 1:**
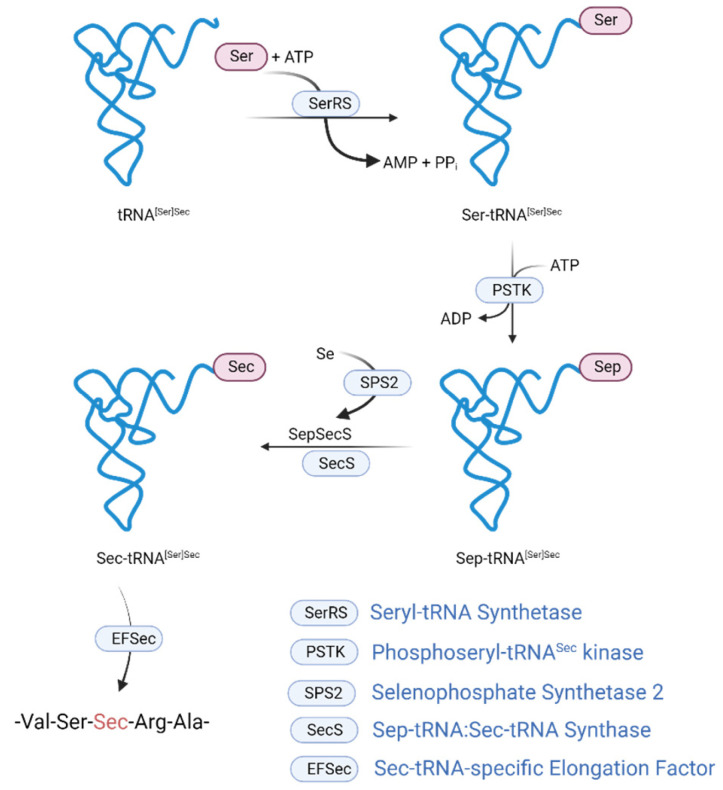
Mechanism of Sec biosynthesis in eukaryotes; tRNA^[Ser]Sec^ is first aminoacylated with serine in a reaction catalyzed by seryl-tRNA synthetase (SerRS) to form Ser-tRNA^[Ser]Sec^ followed by Ser phosphorylation catalyzed by PSTK. SPS2 ensures the synthesis of the selenium donor, selenophosphate. Further, SecS catalyzes Sec-tRNA^[Sec]^ formation. EFSec elongation factor binds the Sec tRNA^[Sec]^ and promotes Sec incorporation in the elongation of the protein polypeptide chain by the ribosome at the UGA codon. Created with BioRender.com (accessed on 19 September 2023).

Sec is inserted into newly synthesized polypeptide chains in response to the UGA codon in a cotranslational manner. The UGA codon is decoded as Sec by using Sec insertion machinery, which requires a cis-acting Sec insertion sequence (SECIS) element [2]. In eukaryotes, Sec is a unique, known amino acid among all the amino acids whose biosynthesis needs its own tRNA, Sec tRNA^[Ser]Sec^ [3,4]. tRNA^[Ser]Sec^ is first aminoacylated with serine in a reaction catalyzed by seryl-tRNA synthetase (SerRS) to form seryl-tRNA^[Ser]Sec^ (Figure 1). tRNA^[Ser]Sec^ acts both as the key molecule and central component [3] of selenoprotein biosynthesis [5].

Regarding selenoproteins and selenoproteomes, there has been significant progress in their characterization, discovering their physiological roles and functions related to a large number of diseases, both in humans and animals. In total, 25 selenoprotein genes have been identified in humans as having quite diverse properties and functions and several are broadly classified as antioxidant enzymes.

In this review, we discuss current knowledge only about the selenoproteins that are named after an alphabetical letter, which are referred to as the “alphabet selenoproteins” and are less known compared to the rest of the selenoproteins.

## 2. “Alphabet” Selenoproteins Structures

Mammalian selenoproteins are classified into two large groups in accordance with their Sec location [6]. One of these two groups contains Sec in a site adjacent to the COOH-terminal region of the protein, such as selenoproteins S, R, O, I, K, and TrxRs (thioredoxin reductases). The other group has Sec in the NH_2_-terminal region of the protein, such as H, M, N, T, V, W, F (Sep15), SPS2 (selenophosphate synthetase 2), GPxs (glutathione peroxidases), and DIOs (deiodinases); most of these selenoproteins have a thioredoxin fold structure and some others contain a C-x-x-U structural motif (U is Sec) according to the thioredoxin active-site C-x-x-C structural motif [6,7,8]. The structure of most selenoproteins suggests their functions in redox-type reactions.

### 2.1. Selenoprotein I (SELENOI)

SELENOI is a multi-pass ER transmembrane protein found only in vertebrates and is a recently evolved selenoprotein [6]. It contains a CDP-alcohol phosphatidyl transferase domain, which is highly conserved and is present in choline (CHPT1) and choline/ethanolamine (CEPT1) phosphotransferases. These two enzymes catalyze the last step of the Kennedy pathway for the synthesis of two phospholipids, phosphatidylethanolamine (PE) and phosphatidylcholine (PC), by transferring phosphoethanolamine and phosphocholine groups from CDP-ethanolamine, and respectively, CDP-choline, to 1,2-diacylglycerol (DAG). CHTP1 catalyzes the synthesis of phosphatidylcholine from CDP-choline, while CEPT1 is not a selenoprotein and can synthesize both phosphatidylcholine and phosphatidylethanolamine. Both enzymes have specificity for CDP-choline and CDP-ethanolamine. SELENOI uses only CDP-ethanolamine as a substrate [9]. The major structural difference between SELENOI, CHPT1, and CEPT1 is the presence of the COOH-terminal extension, which contains the Sec residue, and whose function is currently unknown. SELENOI contains, similar to choline phosphotransferases, seven transmembrane domains and three conserved aspartic residues that are involved in the catalytic activity within a DG(X)_2_AR(X)_8_G(X)_3_D(X)_3_D structural motif [10].

### 2.2. Selenoprotein O (SELENOO)

The Human selenoprotein, O, is poorly characterized. Homologs of mammalian selenoprotein, including human SELENOO, were found in a wide category of organisms, including plants, bacteria, and yeast. In mammals, it is located in mitochondria, and the C-x-x-U structural motif in human SELENOO suggests that it, like other selenoproteins, could engage in redox activity interaction with a protein through this structural motif. This conclusion is supported because experiments have shown that the S-x-x-C motif, in which Cys was replaced with Ser, enabled the mutant SELENOO to trap a redox protein target. The S-x-x-S or C-x-x-S structural motifs prevented the formation of an interprotein disulfide bond [11]. Moreover, the effect of varying the H_2_O_2_ concentrations on the formation of a protein complex involving S-x-x-C and S-x-x-S mutant forms of SELENOO has shown that the protein complex was formed only in the case of S-x-x-C and not of S-x-x-S. These experiments demonstrated that human SELENOO interacts in a redox type with a target protein through the conserved C-x-x-U motif and that this interaction is reversible and dependable on H_2_O_2_ treatment as a specific property of thiol-dependent oxidoreduction. Recently, a hypothesis emerged that SELENOO may have a kinase function [11,12,13].

### 2.3. Selenoprotein N (SELENON)

SELENON, encoded by the *SEPN1* gene, was identified as an ER integral membrane protein [14].

All the experiments revealed that selenoprotein N is a type II transmembrane protein having a single transmembrane domain in the NH_2_-terminal region. The examination of the amino acid sequence using bioinformatics tools predicted that four putative N-glycosylation sites are present at positions Asn156, Asn449, Asn471, and Asn497. Selenoprotein N translocation across the ER membrane can be demonstrated using any one of these sites, as the active site of the oligosaccharyltransferase complex is located in the ER lumen. In order to search for known domains within the luminal sequence using bioinformatics tools, this analysis predicted the presence of a sequence that resembles the calcium-binding domain, known as EF-hand, together with a thioredoxin (Trx) domain containing the selenocysteine residue.

Usually, EF-hand has a helix–loop–helix structure in which the two α-helices allow calcium ion coordination in a pentagonal bipyramidal configuration [15]. Structure prediction of selenoprotein N EF-hand using the QUARK algorithm (https://zhanglab.ccmb.med.umich.edu/QUARK/, accessed on 25 June 2023) reveals a single α-helix followed by a flexible loop and a β-sheet structure instead of the second α-helix. Moreover, despite the high sequence similarity to an EF-hand, the predicted EF-hand of SELENON may have an uncommon structure. This fact raised the question of whether this uncommon structure of the EF-hand domain is functional and able to bind calcium. Experiments performed on generated synthetic peptides of 36 amino acids representing a wild-type (WT) selenoprotein N EF-hand versus three mutants with a single amino acid substitution revealed only for WT, significant calcium-dependent conformational changes, and not for the mutant versions. In addition, the WT peptide binds calcium with a constant affinity in the concentration range of ER calcium [16].

### 2.4. Selenoproteins H, T, V, and W (SELENOH, SELENOT, SELENOV, and SELENOW)

These selenoproteins belong to the thioredoxin-like (Rdx) family of selenoproteins and thus contain a thioredoxin-like fold with a characteristic and conserved Cys-x-x-Sec structural motif. Due to these facts, the Rdx family can be considered thiol-based oxidoreductases, but their exact functions remain unknown. In addition, they possess a conserved chain of amino acids at the COOH-terminal region with the tGxFEI(V) consensus sequence.

SELENOW is a small 9-kDa cytosolic protein, and in mammals, it is one of the most abundant selenoproteins. It is one of the first identified selenoproteins and is mainly expressed in muscle and the brain [17,18]. The expression of SELENOW is highly regulated by the availability of Se in the diet, being part of the stress-related group of selenoproteins [19]. Experiments found that selenoprotein W purified from rat muscle can form a complex with glutathione [20]. Another target of the mammalian SELENOW is represented by the 14-3-3 protein family, originating only from eukaryotes and having a ubiquitous expression that specifically binds a distinct phosphoserine or phosphothreonine motif to accomplish their effects on their target proteins, such as conformational change or other structural protein features [7]. Using NMR spectroscopy, two flexible external loops in selenoprotein W were identified to be involved in binding 14-3-3 protein, but the deep molecular mechanisms of the regulation of 14-3-3 protein by SELENOW remain unknown. However, two hypotheses emerged regarding the SELENOW implication. So, SELENOW could be involved in the redox regulation of 14-3-3 protein [21] and/or in the interaction of 14-3-3 protein with its binding counterparts CDC25B [22] and Rictor [23].

Selenoprotein T (SELENOT) is one of the first selenoproteins identified using bioinformatic tools [24]. SELENOT is a thioredoxin-like protein and it is highly expressed during the development of the embryo. In adults, it is retained in endocrine tissues, where its function remains unknown [7]. Selenoprotein T is expressed, predominantly, at the ER membrane of all pituitary cells and also at the Golgi complex [25]. The loss of SELENOT determines the upregulation of several oxidoreductase genes, such as SELENOW, which is another member of the Rdx family [2].

Selenoprotein H (SELENOH) is a 14-kDa selenoprotein, has a Sec residue within the Cys-x-x-Sec structural motif, and is localized specifically to the nucleoli. It also contains a conserved nuclear RKRK motif in the NH_2_-terminal sequence [26]. Selenoprotein H is dependent on dietary selenium intake, similar to selenoprotein W [19]. SELENOH contains an AT-hook motif, also present in DNA-binding proteins. Using an in vitro chromatin immunoprecipitation assay, it was shown that SELENOH binds specifically to DNA sequences corresponding to heat shock and stress response elements [27].

Selenoprotein V (SELENOV) is a less characterized selenoprotein that evolved recently, probably by duplication of selenoprotein W. SELENOV is localized in mammal placenta and testes [10]. Selenoprotein V is similar to selenoprotein W but is larger due to the presence of an additional NH_2_-terminal region, with an unknown function, which is not present in selenoprotein W. SELENOV detected in testes may be involved in male reproduction function but its exact function is still unknown.

Chen et al. showed that SELENOV knockout in mice led to changes in mRNA levels, protein amounts, and enzyme activities of selenoproteins Gpx1, TrxR1, and SELENOP. SELENOV mRNA and SELENOV protein itself were undetectable in the liver and were unaffected by dietary selenium in the testis; the SELENOV knockout resulted in decreased selenium concentrations in the liver, Gpx activities in the serum and liver, Gpx1 activity, and protein amounts in the testis, and mRNA of Gpx1, SELENOP and/or TrxR1 in the testis and/or liver, suggesting the potential major role of SELENOV in selenium homeostasis. In addition, decreased SELENOP levels had an essential impact on the expression of other selenoproteins [28].

### 2.5. Selenoprotein M (SELENOM) and the 15-kDa Selenoprotein of the Selenoprotein M Family (Sep15, Selenoprotein F, and SELENOF)

Selenoprotein M and SELENOF (Sep15) form a distinct selenoprotein family localized in ER and are thioredoxin-like fold proteins. Selenoprotein M has high expression in the brain. Sep15 is highly expressed in the liver, kidney, prostate, and testis. SELENOM was identified using bioinformatic tools [29].

Sep15 and selenoprotein M have 31% sequence identity, and both have a common thioredoxin-like domain and contain an NH_2_-terminal signal peptide. SELENOM has a COOH-terminal extension that has an ER retention signal, in contrast with Sep15, which lacks it. Sep15 possesses a distinct Cys-rich domain in the NH_2_-terminal region of the protein, which is needed for the interaction of Sep15 with UDP-glucose-glycoprotein glucosyltransferase (UGGT), which is the binding compound of Sep15 [30]. The fact that Sep15 lacks the ER retention signal suggests that this selenoprotein owes its binding to UGGT for its maintenance in the ER rather than through mediating by that specific ER retention signal.

Using NMR spectroscopy, the structures of the thioredoxin-like domain of Sep15 in Drosophila and of selenoprotein M in mice have been solved [8]. Both proteins have an α/β-fold, which is common for thioredoxin-like oxidoreductases and includes a four-stranded β-sheet structure that is surrounded by three α-helices. Both contain redox-active motifs within a loop situated between strand β1 and helix α1, with these structural motifs being similar to other oxidoreductases that contain thioredoxin-like reductase, suggesting their oxidoreductase function. At the same time, there are differences from other oxidoreductases, such as thioredoxin and protein disulfide isomerase, regarding the consensus sequences of the redox motifs C-x-U in Sep15 and C-x-x-U in selenoprotein M. It is to be mentioned that the COOH-terminal extension of selenoprotein M that is lacking in Sep15 is very flexible, indicating the plausible role of this flexible region in the binding of the substrate or the interaction with other protein factors. In addition, the presence of redox-active structural motifs and the structure similarity to other thioredoxin-fold oxidoreductases are suggestive of the possible function of Sep15 and SELENOM in catalyzing the reduction of disulfide bonds in the ER proteins or other secretory proteins [31].

Studies have shown that Sep15 tightly forms a complex, in a 1:1 ratio, with UGGT [30]. UGGT is a chaperone implicated in the regulation of the calnexin (CNX) cycle. This cycle represents a pathway for controlling the adequate quality of proteins and supervises the accurate folding of N-linked glycoproteins in the ER [32,33]. Sep15 may have roles in the formation of disulfide bonds and in the quality control of the group of glycoproteins that are UGGT substrates [31]. Moreover, Sep15 expression is induced when misfolded proteins are accumulated in the ER [34]. The expression of Sep15 is dependent on dietary selenium; however, under selenium deficiency, the decrease in Sep15 in the brain and the testis is not a very marked one [8,35]. Sep15 has also been included in the stress-related selenoproteins group. An important structural fact regarding the human *Sep15* gene is that it has been revealed the presence of two multimorphic sites at nucleotide positions 811 (C/T) and 1125 (A/G), the latter one being located in the SECIS element, that influence the efficiency of Sec incorporation into the *Sep15* gene in a Se-dependent way [36,37,38]. Moreover, it was described that among African Americans, the A1125 variant is the most frequent and it may be associated with an increased incidence of tumors in the breast, head, and neck. Different studies have demonstrated that Sep15 is involved in preventing liver [36], prostate [38], breast [38], and lung cancers [39]. In contrast, other studies demonstrated that Sep15 is implicated in promoting some types of cancer, including colon cancer [40,41].

### 2.6. Selenoproteins K (SELENOK) and S (SELENOS)

Even though these two selenoproteins have no significant sequence similarity, they form a single family. The characteristics that these selenoproteins share for assigning them as a family of related selenoproteins are the following: a single transmembrane domain in the NH_2_-terminal region; the presence of a glycine-rich (G-rich) sequence with an exceptionally high content of glycine, proline, and amino acids with a positive charge; and a specific localization of Sec residues in the COOH-terminal region of the protein [42].

Genomics studies have shown that the Sec-containing SELENOK/SELENOS-like protein family is the most frequently encountered eukaryotic selenoprotein family, including humans, whereas, in fungi, insects, and plants, Cys-containing SELENOK/SELENOS-like proteins were identified.

SELENOK and SELENOS are transmembrane type III proteins, which are localized to the ER membrane. They contain a single transmembrane domain, with the COOH-terminal region highly unstructured and containing the glycine-rich (G-rich) domain, situated in the cytoplasm. In addition, the homologs of selenoproteins K and S contain Sec in the third or second position from the COOH-terminal region. This group of selenoproteins is different from the ER selenoproteins containing thioredoxin-like fold.

The recent experiments worked out using recombinant techniques for solving the structure of the cytosolic region of human selenoprotein S (cSELENOS), in which Sec188 was replaced by Cys, revealed that the cytosolic end portion of the SELENOS contains two extended α-helices that are followed by an exceedingly disordered COOH-terminal region [43]. This disordered nature of the C-terminal region may suggest that this region can be flexible in order to interact with many structurally different substrates, such as, for example, misfolded proteins. In addition, this cytosolic end portion of cSELENOS contains a disulfide bond between Cys174 and Cys188, suggesting the formation of a selenylsulfide bond in the native selenoprotein S. Recent studies succeeded in biochemically characterizing a Sec-containing cSELENOS [44,45]. Moreover, these findings confirmed the presence of the selenylsulfide bond, and so a probable reductase function for selenoprotein S. The −234 mV redox potential of SELENOS may indicate that SELENOS could be implicated in the ERAD substrates’ disulfide bond reduction before the translocation from the ER using the Trx/TR system as the electron donor [44].

Genetic variations of SELENOS in humans are associated with a large number of diseases based on the fact that SELENOS is implicated in numerous signaling pathways. A single-nucleotide polymorphism located in the ER stress response element led to high levels of proinflammatory cytokines, such as IL-6, Il-1β, and TNFα [46]. Polymorphisms increase the Kashin–Beck Disease [47], Hashimoto’s thyroiditis [48], colorectal cancer [49,50,51], and gastric cancer [52]. SELENOS is upregulated in Crohn’s Disease [53] and after focal cerebral ischemia [54]. Increased expression of selenoprotein S determines a decrease in tau protein phosphorylation in Alzheimer’s Disease (AD) [55]. These genetic polymorphisms of SELENOS have proved to be associated with risk factors for diabetes mellitus, inflammatory processes, dyslipidemia, cardiovascular diseases, and cancer. High expressions generally correlate with poor prognostic in some types of cancer, such as gastric and thyroid cancer [56].

### 2.7. Selenoprotein P (SELENOP, Sepp1)

Selenoprotein P was described for the first time in 1973 by Burk et al. [57] and Rotruck et al. [58]. SELENOP is an extracellular monomeric 41 kDa (but migrates in multiple bands of 50–60 kDa in SDS-PAGE, probably due to variations in glycosylation [59]) secreted glycoprotein and the homologs of SELENOP are found mostly in vertebrates. The “P” originated from the presence of SELENOP in plasma. Selenoprotein P contains 10 Sec, 17 Cys, and 14 His residues. It is an abundant selenoprotein that accounts for almost 50% of the total Se in plasma [60].

SELENOP has two domains. The NH_2_-terminal domain is the larger one, it represents two-thirds of the amino acid sequence and contains one selenocysteine residue (U) in a U-x-x-C redox structural motif. The second domain, the COOH-terminal one, is smaller and contains multiple Sec residues, for example, a number of 9 Sec residues in rats, mice, and humans [61]. These multiple Sec residues are a unique feature of SELENOP among other selenoproteins. These facts that selenoprotein P is secreted into the plasma, where is abundant and contains most of the selenium, and these multiple Sec residues in its sequence, suggested that SELENOP might have the function to supply selenium for tissues [62].

The N-terminal domain contains conserved sequences similar to bacterial thioredoxin-fold proteins, suggesting that this domain has a thiol-redox function and that selenoprotein P is a member of the thioredoxin superfamily [60,63,64,65].

Regarding the COOH-terminal domain, in marine animals, SELENOP is evolved, and it contains 27 Sec residues, probably due to their necessity to supply selenium for their great number of selenoproteins, whereas terrestrial animals have fewer selenoproteins and therefore their SELENOP molecules have fewer Sec residues as a consequence, presumably because the most selenocysteine residues are replaced with cysteine residues.

Moreover, Lobanov et al., using bioinformatic analysis, demonstrated that the two domains of selenoprotein P also have distinct functions. They analyzed the bioinformatics of the selenoproteome using the techniques BLASTP (Basic Local Alignment Search Tool), TBLASTN (which compares a protein query sequence to all six possible reading frames of a database in order to identify proteins in new genomes), and PSI-BLAST (Position-Specific Iterative-BLAST, a protein sequence profile search method that builds off the alignments generated by a run of the BLAST program) [61].

Selenoprotein P binding to heparin is dependent on pH values [66,67,68]. Heparin binds to a heparin-binding site situated at the NH_2_-terminal domain that contains histidine as two of its five basic residues [69]. Only when pH has values below the physiological range does histidine become protonated and able to bind heparin. SELENOP binds to heparin only under acidic conditions and remains unbound at physiologic pH.

The NH_2_-terminal domain of SELENOP contains two histidine-rich stretches containing a sequence of up to 10 basic amino acids which probably are involved in the heparin-binding, a characteristic property of selenoprotein P [69,70]. The NH_2_-terminal domain also contains carbohydrates with three occupied N-glycosylation sites; the COOH-terminal domain contains one occupied O-glycosylation site. In the purified rat SELENOP structure, several disulfide and selenylsulfide bonds have been identified, which might have structural functions and also might protect the selenium atoms by reducing their activity [71]. Purified SELENOP from rat plasma has four isoforms. The full-length isoform has 10 Sec residues, whereas the shorter isoforms terminate at the second, third, and seventh selenocysteine positions, containing 1, 2, and 6 Sec residues, respectively [72,73].

SELENOP is not a homogenous protein, its three-dimensional structure has not been resolved, and its characterization was accomplished on the endogenous protein using cell lines or by purifying SELENOP from plasma and serum [74].

The literature reported studies concluded that the plasma concentrations of selenoprotein P in healthy subjects have large variations between 0.2 and 6 μg/mL, probably because of yet unstandardized methods and the variations of selenium intake [59].

The liver is the major source of SELENOP for plasma where its turnover is rapid. The liver secretes highly glycosylated selenoprotein into plasma. Selenoprotein P is also expressed in mammalian species in other tissues including the testes, brain, and gut, with SELENOP mRNA expression having been detected in virtually all tissues.

The mRNA for selenoprotein P has the unique feature of containing 10 UGA codons in the open reading frame (ORF) and 2 SECIS in the 3′UTR whereas mRNA of other selenoproteins contains only one SECIS element [75]. Those 10 Sec residues are involved in the SELENOP function: one Sec residue, at the NH_2_-terminal region, forms an active site of enzyme activity to reduce phospholipid hydroperoxide while the other nine COOH-terminal Sec residues are implicated in the selenium transportation [76]. SECIS 2 is less efficient than SECIS1. The first SECIS element is localized on the 5′ side near the stop codon and facilitates the processive Sec incorporation while the second SECIS element has the role of slow decoding at the first UGA codon. SECIS 2 was shown to be designated for the insertion of the single Sec residue in the NH_2_-terminal region, and this process takes place slowly. Once the ribosome passes the second UGA it interacts with SECIS 1 which acts fast and inserts the other remaining Sec residues [76,77].

**Figure 2:**
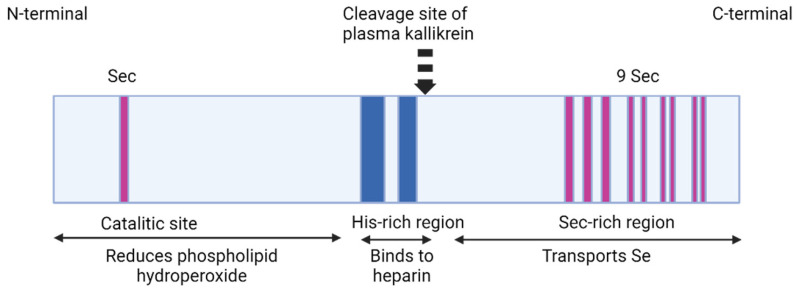
Selenoproteins P—schematic structure. The figure shows a schematic structure of SELENOP with its N-terminal and C-terminal domains, the His-rich region, Sec residues localization, and SELENOP functions. Created with BioRender.com (accessed on 19 September 2023).

SELENOP is cleaved via limited proteolysis by plasma kallikrein between Arg235-Gln236 and Arg242-Asp243 in an N-terminal fragment (1–235) with a role in the enzyme activity and a C-terminal fragment (235–361) involved in Se-supply activity [76]. The N-terminal domain is presumably a catalytic center of selenoprotein P and possesses a U-x-x-C motif similar to the C-x-x-C motif of the thioredoxin catalytic center, suggesting the specificity for protein thiols. Moreover, SELENOP has an even greater specificity, and it uses, besides GSH, other thiols such as TRX, mercaptoethanol, and dithiothreitol as reducing agents, whereas cellular GPX1 (glutathione peroxidase 1) uses only GSH as a reducing agent [65] (Figure 2).

Experiments conducted in vitro have shown that the amino acids residues 324–326 that are localized between the fifth and the sixth Sec positions, as referred to (Cys-Gln-Cys) sequence, are essential for the binding of SELENOP to apoER2 receptor, and a molecule consisting only the residues 208–361 of the COOH-terminal could also bind to apoER2 receptor [78]. These results are linked with in vivo observation that apoER2 does not mediate the uptake of a form of SELENOP that ends at the second selenocysteine position, and also that the interaction of apoER2 exclusively with the long forms of SELENOP increases the selenium uptake efficiency [79,80]. Moreover, the SELENOP-apoER2 interaction occurs in the β-propeller domain of apoER2, which is characterized by 4 to 8 highly symmetrical blade-shaped β-sheets arranged toroidally around a central axis, and not in the ligand-binding repeat region, which is destined to bind the other apoER2 ligands. All forms of apoER2, even the splice variants, contain the β-propeller domain and are believed to mediate the endocytosis of the COOH-terminal domain of SELENOP [81].

Experiments performed by Hill et al. in 2007 on a mice model have demonstrated that deletion of the C-terminal region of SELENOP had important effects on brain and testis selenium regardless of the amount of selenium administrated, while kidney selenium was not affected when fed with normal nutritional requirement (0.1 mg/kg). At higher selenium dietary levels, the effects on kidneys were not very significant. The researchers concluded that the whole organism and kidneys selenium depend more on the N-terminal region of SELENOP than on the C-terminal region, while the brain and the testis depend more on the C-terminal domain. Mice with deletion of the C-terminal domain have neurological and male reproductive phenotypes similar to those lacking the whole protein SELENOP. The N-terminal domain has essential roles in the conservation of the whole body selenium and may supply selenium to kidneys [80].

## 3. Signaling Pathways Mediated by “Alphabet” Selenoproteins

### 3.1. Signaling Pathways That Are Mediated by the Endoplasmic Reticulum (ER) Membrane Selenoproteins (SELENON, SELENOK, SELENOT, SELENOI)

**Figure 3:**
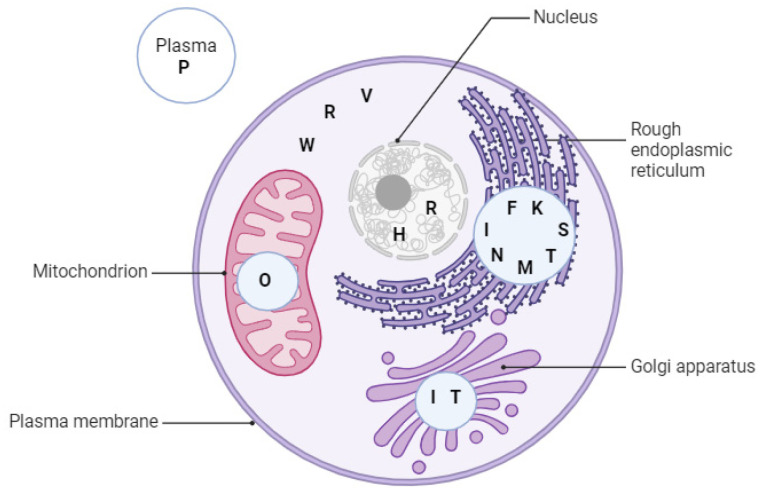
Subcellular localization of “alphabet” selenoproteins; H and R are localized in the nucleus; O in mitochondria; I and T in Golgi apparatus; F, I, K, M, N, S, and T in ER; R, V, and W in cytoplasm and P in plasma. Created with BioRender.com (accessed on 19 September 2023).

Selenoprotein N, encoded by the *SEPN1* gene, is a transmembrane protein type II and senses ER calcium levels by binding calcium ions through its luminal EF-hand domain. It was found that this domain is essential for the response of SEPN1 when luminal calcium levels are low by changing its oligomeric structure and enhancing its redox cellular intervention, including the interaction with the ER calcium pump sarcoplasmic/endoplasmic reticulum calcium ATPase (SERCA), in order to refill ER calcium stores. SERCA2b, an isoform of SERCA, interacts with the oxidoreductases ERp57 and ERdj5 which modulate the activity of ER calcium reuptake [16,82,83]. Inositol trisphosphate receptor (IP3R) and ryanodine receptor (RyR) have an opposite action from SERCA2b and determine calcium release from the ER [83,84,85,86,87]. ER calcium levels are essential for the correct protein folding and the abrupt gradient across the ER membrane, and are needed for rapid excitation–contraction coupling. SEPN1 protects the ER from peroxides that are produced by ER oxidoreductin 1 (Ero1) which belongs to the oxidoreductases family enzymes, and also protects ER from ER thiol oxidase activity. SELENON stimulates the activity of SERCA2, and SERCA2b reduces the number of cysteines that are intensely oxidized by peroxides generated by Ero1. Marino et al. experiments showed in 2015 that cells lacking SEPN1 become defective upon the calcium ions reuptake in ER due to a hypersensitivity to overexpression of Ero1 that modifies the redox potential of ER to a more oxidized state, and this effect alters calcium uptake [88]. These facts suggest the SEPN1 role in the stress-dependent antioxidant ER response and may explain the pathogenic mechanism of SELENON-related myopathies [89] (Table 1) (Figure 3).

Mutations in the *SEPN1* gene, causing the knockdown of SELENON accompanied by recessive gene *RYR1* that encodes ryanodine receptor 1 (RyR1), which are both proteins implicated in calcium homeostasis, cause severe congenital myopathies. In addition to myopathies, these mutations also lead to impaired insulin action in skeletal muscle by decreasing Akt (protein kinase B) phosphorylation and high ER stress. All these facts indicate a correlation between the decrease in glucose tolerance, insulin activity, and increased ER stress in muscles [90].

**Table 1:** Cellular localization and function of “alphabet” selenoproteins.

Selenoprotein Name	Cellular Localization	Function
Selenoprotein F (Sep15, selenoprotein M family)	ER lumen	Protein folding [91]
Selenoprotein H	Nucleus	Nucleolar oxidoreductase [92] Redox homeostasis [92] Cell cycle regulator [93]
Selenoprotein I	ER membrane/Golgi apparatus	Phospholipid biosynthesis [9,10]
Selenoprotein K	ER membrane	ER responses to stress [94] Ca^2+^-dependent signal transmissions [94] Immune response process [95]
Selenoprotein M	ER lumen	Thiol-disulfide oxidoreductase [96] Calcium homeostasis [97] Hypothalamic signaling via leptin [98]
Selenoprotein N	ER membrane	Calcium signaling [82,83,84,85,86,87] Protects ER from oxidative stress [88] Role in early muscle protein folding [88]
Selenoprotein O	Mitochondria (in mammals)	Probable redox function [11,12,13]
Selenoprotein P	Bound to endothelial cell wall	Selenium transport [59,99] Oxidative stress [65]
Selenoprotein R (MsrB1)	Cytoplasm Nucleus	Methionine sulfoxide reductase [100]
Selenoprotein S	ER membrane and lumen	Anti ER stress effects [101] Antioxidant protection [102] Removal of misfolded proteins [43] Inflammatory responses [103]
Selenoprotein T	ER membrane	Hormone synthesis [104] Calcium mobilization [105] Redox regulation [25]
Selenoprotein V	Cytoplasm of testes and mammal placenta	Testes specific expression [10]
Selenoprotein W	Cytoplasm	Antioxidant role in oxidative stress [19]

The effect of SELENON on the myometrium smooth muscle cells has also been described. In these experiments, using polymerase chain reaction, mRNA levels were quantified on a mouse model and the protein by Western blotting. In addition to those methods, the myometrial tissues were studied via immunohistochemical analysis. The results concluded that when selenium is supplemented to the smooth muscle of the myometrium, the delivery of Ca^2+^ and Ca^2+^-calmodulin complex increases. Moreover, the activity of myosin kinase and phosphorylation of the myosin light chain are stimulated but with no change regarding the amount of reactive oxygen species [106].

Rederstorff et al. described for the first time a mammalian model for SELENON deficiency in *SEPN1* gene knockout mice, in the context of trying to explain the pathophysiological mechanisms implicated in SEPN1-Related Myopathy (SEPN1-RM), a group of muscle diseases with variable severity, caused by mutations in the *SEPN* gene encoding SELENON. The main features of this group of diseases include atrophy and weakness of trunk muscles which start in infancy and lead to severe complications later in life, such as scoliosis and respiratory failure. For SEPN1-RM there is no treatment available so far. Surprisingly, SEPN1 knockout mice were healthy, and they were not distinguishable from the wild-type mice. Rederstorff et al. detected only fine modifications in the morphology, ultrastructure, and contractility of the mutant muscles. The researchers concluded that, in mice, SELENON is dispensable for survival, muscle development, and muscle preservation, under basal conditions. The differences between the absence of basal phenotype in the mice model and the symptoms described in human SEPN1-RM patients might result from the differences in the use of specific muscles, such as postural muscles, which are constantly active for maintaining the vertical position in humans that is distinct situation from mice [107]. Patients with SEPN1-RM have a quasi-normal life before the full phenotype develops in puberty [108], while mice are protected from the development of the phenotype under basal conditions which include a stable environment, restricted physical activity, and controlled conditions of temperature or stress [107].

Studies have revealed that, in regard to selenoproteome, the removal of the selenocysteine tRNA^sec^ gene in knockout mice causes early-stage embryonic lethality, suggesting the important role of selenoprotein synthesis in early mammalian development [109]. Furthermore, a selenoprotein-deficient mouse model was obtained by insertion into the mouse genome of 20 copies of a mutant selenocysteine tRNA^sec^ gene whose product of transcription lacked 6-isopentenyl adenosine, a modified base that is essential for its activity [110]. Overexpression of this mutant tRNAsec interfered with normal selenoprotein synthesis in a specific manner for the protein and tissue [111,112]. It was obvious that these mice presented lower levels of several selenoproteins in different tissues. Studies have shown mice had normal phenotypes regarding skeletal muscle but muscles became heavier after strenuous exercise compared with normal mice, due to an adaptative amplification to effort [113].

Selenoprotein K (SELENOK), which is a transmembrane protein of ER, is involved in ER responses to stress and also in calcium-dependent signaling pathways. SELENOK forms a SELENOK/DHHC6 complex by binding to DHHC6 apoenzyme. This complex accomplishes palmitoylation in target proteins. One of these target proteins is the inositol 1,4,5-trisphosphate receptor (IP3R), so SELENOK is in charge of the stabilization of this calcium channel in the ER membrane. One of the mechanisms that are involved in the activation of immune cells is associated with an increased flow of calcium from the endoplasmic reticulum into the cytosol after inositol-1,4,5-trisphosphate (IP3) binds to its receptor (IP3R) in the endoplasmic reticulum. Experiments revealed that if SELENOK was knocked down, IP3 levels did not decrease but IP3R expression was significantly lowered due to affecting the palmitoylation of IP3R. Immunofluorescence and co-immunoprecipitation techniques demonstrated an interaction between SELENOK and the membrane domain of the enzyme, which is responsible for palmitoylation (DHHC6). If this domain is knocked down, IP3R expression is decreased and consequently the IP3R-dependent Ca^2+^ flux is disrupted [94]. Low SELENOK levels disrupt the calcium flow provided by IP3R. Studies performed by Marciel and Hoffman in 2019 showed that this signaling pathway is involved in the proliferation and activation of immune cells and also in the progression of melanoma. According to research, the expression of SELENOK is required for the development of melanoma because SELENOK is necessary for the flux of Ca^2+^ ions into the cancerous cells. In the experiments, when CRISPR/Cas9 was used to knock down SELENOK in human melanoma, Ca^2+^ flux decreased and impaired the function of receptor IP3R, which inhibited proliferation, invasion, and cell migration. It was concluded that, in the melanoma cell, the tumor growth and its metastatic evolution depend on the SELENOK expression [114].

In regard to the SELENOK role in the immune response process, it has been shown that its knockdown decreases the expression of the CHERP, an ER membrane protein involved in calcium transport, and also reduces the free calcium levels inside the cell. Moreover, the expression of the interleukin-2 receptor alpha chain (IL-2Rα) and interleukin-4 (IL-4) secretion, which play essential roles in T-lymphocyte proliferation and activation, decreases. In their experiments, on a specific cell line, Wang et al. demonstrated that the abovementioned effects are excluded by selenomethionine (Se-Met), which reverses the change in CHERP expression and intracellular free calcium. They used low concentrations of Se-Met that have been shown to increase SELENOK expression, which in turn up-regulated the expression of CHERP and the intracellular free calcium. They concluded that SELENOK may adjust the release of calcium ions by regulating the expression of CHERP. By its actions of regulating CHERP expression, free Ca^2+^ concentration, IL-2R, and IL4 secretion, the endogenous SELENOK plays essential roles in the proliferation and differentiation of T cells [95]. Moreover, Verma et al. revealed in 2011 that the migration of T cells and neutrophils was decreased in SELENOK knock-out mice but the development of the immune system was normal. SELENOK-deficient T cells, neutrophils, and macrophages had a decreased receptor-mediated calcium flux [115].

A high expression of Selenoprotein T (SELENOT) was found, during embryogenesis, in immature tissues. Immunocytochemical assays revealed that SELENOT is localized to the ER through a nonpolar domain.

During PC12 cell differentiation, experiments of Luca Grumolato et al. identified the SELENOT gene as a new gene of interest in the neuropeptide pituitary adenylate cyclase-activating polypeptide (PACAP). PACAP and cAMP induced a rapid and long-lasting increase in SELENOT gene expression in PC12 cells that depends on Ca^2+^ levels. So, overexpression of SELENOT in PC12 cells increased the concentration of intracellular calcium levels dependent on Sec residue while SELENOT knockdown inhibited the PACAP-induced increase in Ca^2+^ and reduced the hormone secretion [105].

SELENOT is also expressed in the ER membrane of all pituitary-secreting cells and has an important role in hormone synthesis because after embryogenesis it remains in endocrine glands such as the pancreas, thyroid gland, testes, and pituitary gland. Moreover, SELENOT’s role in the mechanism of insulin and corticotropin release has also been shown [104].

Deletion of SELENOT in corticotrope cells causes a decrease in hormone production and lowers endoplasmic reticulum-associated protein degradation (ERAD) because the cells promote an unfolded protein response (UPR) and ER stress [25].

Selenoprotein I (SELENOI) is still incompletely characterized. Recent studies demonstrated that SELENOI is mainly localized in the Golgi apparatus [116]. It is widely distributed throughout all tissues with low tissue specificity but it is most abundant in the brain with low regional specificity. That corresponds to the ubiquitous distribution of phosphatidylethanolamine (PE) and plasmenyl phosphatidylethanolamine (plasmenyl PE) in the body, having structural functions in cellular membranes.

SELENOI belongs to two different protein families: one is the selenoprotein family, which has a redox reactive Sec residue, and the other one is the lipid phosphotransferase family, which contains the highly cytidine diphosphate (CDP)-alcohol phosphotransferase motif. In the interface of the cytosol and ER, SELENOI catalyzes the third step, the last one, in the Kennedy pathway for the synthesis of the phospholipid PE [117]. SELENOI is implicated in the de novo synthesis of PE and plasmenyl PE. Plasmenyl PE is also a phospholipid, an ether-linked plasmalogen, that results from the transfer of phosphoethanolamine from cytidine diphosphate (CDP)-ethanolamine to 1-alkyl-2-acylglycerol (AAG). Plasmenyl PE is synthesized through another separate pathway which begins in peroxisomes and finishes in the ER membrane. SELENOI catalyzes the sixth reaction of seven which is comprised of this pathway [118].

Mouse models have been used to discover how selenoproteins are involved in brain development and function. Animal knockout model studies have revealed embryonic lethality for Gpx4, TrxR1, TrxR2, SELENOT, and SELENOI [119]. Avery et al. demonstrated, in their experiments, that SELENOI homozygous deletions in mice lead to early embryonic lethality. Its lack of expression abolishes the development before uterine implantation of embryos on E6. They concluded that SELENOI-dependent adequate phosphatidylethanolamine levels may be needed, during implantation, for membrane interactions between embryo and uterus and SELENOI deficiency hinders cell cycle progression past the blastocyst stage [120].

Mutations in the human SELENOI gene, found in rare cases, lead to a form of hereditary spastic paraplegia (HSP). HSP is an upper motor disease characterized by spasticity of the lower limbs. Other symptoms include ataxia, cognitive/intellectual impairment, impaired vision/hearing, and seizures [121,122].

Recent studies revealed the essential roles of SELENOI in T cell functions by also using SELENOI knockout models. In varied immune responses, the upregulated SELENOI, a consequence of the activation of TCR (T-cell receptor), determines the proliferation and differentiation of T cells. SELENOI has been identified as an anabolic enzyme implicated in metabolic reprogramming. Experiments performed on T cells in SELENOI knockout mice models have shown that its loss of activity protected mice from multiple sclerosis (MS) and experimental autoimmune encephalitis (AEA) by decreasing Th17 pathology [118]. Moreover, the synthesis of ethanol phospholipids has been demonstrated to play a very important role during T cell activation, and in metabolic reprogramming in pluripotent stem cells and tumor cell proliferation [118,123,124].

### 3.2. Signaling Pathways Mediated by the Endoplasmic Reticulum (ER) Lumen Selenoproteins (SELENOF, SELENOS, SELENOM)

Selenoprotein S (SELENOS) gene deletion in preadipocytes causes cell death via an impaired function of the most important regulator of apoptosis, Bcl-2, which controls the permeability of the mitochondrial membrane. SELENOS knockdown increases the level of the essential protein that modifies gene expression during ER stress, the IRE1α protein (inositol transmembrane kinase/endoribonuclease 1α, which is a modulator of gene expression) and p-JNK (Jun N-terminal kinases) implicated in apoptotic signaling, and at the same time, SELENOS knockdown lowers XBP1 (Xbox-binding protein), a transcription factor involved in regulating the expression of genes for an adequate function of the immune system and cellular response to stress. All these facts suggest that SELENOS promotes cell survival through the IRE1α-XBP1 signaling pathway [101].

Li et al., in 2018, studied the effect of the knockdown of SELENOS during the action of reactive oxygen species (ROS) on apoptosis and necrosis using a mouse hepatoma model. The results showed impaired intracellular calcium homeostasis, ROS accumulation, mitochondrial functional disorder, and ATP loss, causing apoptosis and necrosis, even in the absence of ER stress. These facts suggest that SELENOS knockdown initiates apoptosis and necrosis in cells by influencing calcium homeostasis, involving ROS-mPTP-ATP in the transformation of cell apoptosis to cell necrosis and consequently increasing the damage [102].

Overexpression of SELENOS has been obtained in a model of human embryonic kidney and mouse neuroblastoma cultured cells by forcing the ER stress. This effect is bound to a mechanism of interaction between membrane protein p97(VCP) and two essential residues, Pro178 and Pro183, of SELENOS for ER-associated degradation [125].

In separate endothelial cells that are subdued to tumor necrose factor (TNF), the expression of SELENOS increased and that fact leads to increased nitric oxide synthase activity and nitric oxide levels. The excess of SELENOS expression blocked the TNF-induced adhesion of THP-1 cells (human leukemia monocytic cell line) to endothelial cells, so managing the involvement of interleukin-1 and interleukin-6 inflammatory factors. In contrast, the knockdown of SELENOS has opposite effects [126].

Ochratoxins, which are mycotoxins produced by some Aspergillus species and some Penicillium species, especially *P. verrucosum*, induce cytotoxicity and apoptosis, which are suppressed via overexpression of SELENOS, and its overexpression increases the level of glutathione, decreases the reactive oxygen species, and inhibits the ochratoxin-induced phosphorylation. The knockdown of SELENOS expression has opposite effects [127]. It has been shown that ochratoxin A (OTA), is the most abundant and the most nephrotoxic, immunotoxic, hepatotoxic, and teratogenic type of ochratoxins to humans and several species of animals. Even if its mechanism of action is not fully elucidated, OTA activates both MAPK/ERK (mitogen-activated protein kinases/extracellular signal-regulated kinases) and PI3K/Akt (phosphoinositide 3-kinase/Akt kinase) signaling pathways implicated in apoptosis and cell survival [128,129].

Regarding inflammation-induced calcification of smooth muscle cells, under a SELENOS knockdown state, when comparing osteoblastic differentiation and calcification of smooth muscle cells, it was found that the lipopolysaccharide activates both classical and alternative nuclear signal transduction pathways-κB (NF-κB) during calcification. Moreover, SELENOS knockdown enhances lipopolysaccharide-induced proinflammatory cytokines and the expression of TNF-α and interleukin-6. SELENOS can suppress inflammation-induced calcification by inhibiting the NF-κB signaling pathway and ER stress [103].

The interaction of SELENOS with the valosin-containing protein (VCP) of the ER membrane manages to insert SELENOK into the ER membrane [90].

Selenoprotein M (SELENOM) is highly expressed in the brain and is known as a thiol-disulfide oxidoreductase, which is involved in disulfide bond formation, and there is a strong bond between the levels of dietary selenium and SELENOM [96]. This selenoprotein is also implicated in calcium and redox homeostasis. SELENOM knockdown increases the calcium baseline levels in the cells, decreases the cell viability (often cells exhibiting blebbing that may indicate apoptosis), and increases the production of reaction oxidative species (ROS) suggesting an essential preventing role in oxidative stress [96,97].

Pitts et al. found that SELENOM-knockout mice exhibited normal brain development with no significant changes in motor coordination, anxiety-like behavior, and cognitive function. At the same time, mice showed an important elevated body weight with increased fat deposits over time compared with wild-type controls. Moreover, mice had elevated serum leptin levels and decreased sensitivity to leptin in the arcuate hypothalamus with glucose tolerance comparable with the wild-type when fed either a normal or a selenium-deficient diet [130].

SELENOM is involved in hypothalamic signaling via leptin, as in vitro and in vivo studies have shown. In the hypothalamus, leptin promotes the SELENOM expression; however, knockdown of SELENOM blocks the phosphorylation leptin dependent on STAT3, which is a protein implicated when a cell is responding to signals via interleukin and growth factor receptors. Overexpression of SELENOM enhances leptin sensitivity. In general, SELENOM proves to be a positive regulator of leptin signaling and of thioredoxin antioxidant activity in the hypothalamus [98].

Studies have shown that (Sec to Cys)-mutant SELENOM and the His-rich domain of SELENOP can bind transition metal ions, and, for example, can modulate Zn^2+^-mediated amyloid-β (Aβ42) aggregation, generation of reactive oxygen species, and neurotoxicity. In neurons, aggregation, and cytotoxicity of amyloid-β peptide with transition metal ions are involved in the progression of Alzheimer’s disease. Aβ42 fibrillization is triggered by binding Zn^2+^ but the fibrillization and also the Zn^2+^ toxicity may significantly be blocked by the His-rich domain of SELENOP and (Sec to Cys)-mutant SELENOM as was confirmed via electronic microscopy and fluorescence assays. Moreover, it is interesting to mention that both these selenoproteins inhibited Zn^2+^-Aβ42-induced neurotoxicity and the production of reactive oxygen species (ROS) in the cells [131].

Overexpression of SELENOM activates the PI3K/Akt/mTOR pathway, an essential signaling intracellular pathway that is involved in cell growth, cell proliferation, metabolism, and avoiding apoptosis. These facts have been shown while studying renal cell carcinoma proliferation and metastasis. Moreover, the proliferated cells reveal an increase in N-cadherin, β-catenin, and vimentin, known to be correlated with tumor progression, metastasis, and cancer prognosis, being, at the same time, markers of the epithelial–mesenchymal transition [132]. Also, an increase in the level of malignancy has been gradually revealed in hepatocellular carcinoma depending on the overexpression of SELENOM detected in liver cells [96].

Selenoprotein F, initially named the 15 kDa selenoprotein (Sep15), is involved in protein folding. SELENOF knockdown using shRNA inhibits cell proliferation and blocks cells in the G1 phase (suppresses cell growth) and ER stress occurs. Moreover, adhesive cell junctions shift into the basal part of the cell, so the invasive and migration abilities are consequently reduced. These events are reversed by restoring the SELENOF levels [91].

Performing an ultramicroscopic examination, Bang et al., in 2015, concluded that if the SELENOF concentration is insufficient, membrane vesicles appear and an increased amount of such endosomes leads to cell shape transformation from spindle to round, and actin fibrils are shifted to the periphery and alpha-tubulin overlaps. All these changes are reversed and inhibited by rho-associated protein kinase inhibitors. It is to be mentioned that cells with SELENOF deficiency are not apoptotic despite the vesicle formation and the arrangement of F-actin and tubulin is an exact one. The studies reveal that SELENOF acts as a pathway regulator that counteracts RhoA/ROCK/MLC-dependent vesicle formation [133].

Overexpression of SELENOF occurs through activation of heat shock and the overexpression of HSF1 (heat shock transcription factor 1) as well as sodium selenite treatment on a Hek293 cell model. Generally, both overexpression and knockdown of HSF1 demonstrate the influence of HSF1 gene transcription regulation on SELENOF and its influence on selenotranscriptome [134].

### 3.3. Signaling Functions Mediated by Selenoproteins of the Cell Nucleus (SELENOH, SELENOR)

Nuclear SELENOH was recently described and acts as a nucleolar oxidoreductase. It is involved in redox homeostasis and can prevent at some level DNA damage [92]. Studies have concluded that SELENOH plays an important role in both redox homeostasis and carcinogenesis. A high expression occurs in tumor tissue and also in undifferentiated tissues in the stomach and intestine epithelium. The silencing of SELENOH leads to a decrease in cell differentiation and stimulates proliferation and migration. Moreover, this silencing promotes the colonization of tumors and xenografts. In addition to the control of proliferation, SELENOH is also a cell cycle regulator [93].

SELENOH has glutathione peroxidase activity and studies have shown that it is involved in the regulation of the transcription of a group of genes that are implicated in de novo glutathione synthesis and phase II detoxification enzymes [26].

### 3.4. Signaling Pathways Mediated by Cytoplasm Selenoproteins (SELENOR)

Selenoprotein R (SELENOR, MsrB1) is a mammalian selenoprotein, an antioxidant enzyme, and has a methionine sulfoxide reductase function. It is localized both in the cytoplasm and nucleus. SELENOR reduces Met sulfoxide produced by oxygen reactive species (ROS) back to Met (methionine) [100]. There are two types of methionine sulfoxide reductases (Msrs): MsrA and MsrB. The MsrB family contains three enzymes, SELENOR (MsrB1), MsrB2, and MsrB3 [135]. It appears that SELENOR evolved separately, so it has little homology with other Msrs and it is found specifically in vertebrates [136]. As Met sulfoxide has two diastereomeric forms, Met-S-sulfoxide (Met-S-SO) and Met-R-sulfoxide (Met-R-SO), the MsrB family reduces Met-R-SO and MsrA reduces Met-O-SO [137,138]. SELENOR has a low catalytic efficiency and, like the other oxidoreductases, requires the recycling of Trx/TrxR/NADPH oxidized form to the reduced form [139]. SELENOR is also implicated in repairing oxidized proteins in order for proteins to maintain their structure and function under oxidative stress conditions [140]. SELENOR is involved in the reduction of oxidized 44 and 47 Met residues in F-actin, preventing its depolymerization caused by MICAL proteins’ oxidation action [141]. In addition, selenoprotein R could interact with many other proteins, such as transient receptor potential channel proteins and β-amyloid proteins. It may have essential roles in the central nervous system and is intensely related to the development of neurodegenerative diseases [142]. SELENOR also acts against apoptosis triggered by oxidative stress in the human lens epithelium and attenuates cataracts [143]. Its action is based on the fact that membrane-bound proteins in the epithelial cells of patients with cataracts contain high levels of Met-R-SO, the substrate for SELENOR [144]. Another role of SELENOR is in innate immunity but the exact mechanism of its action is still to be discovered. In the macrophages, its expression is induced by lipopolysaccharides and controls macrophage function by promoting the expression of anti-inflammatory cytokines, such as IL-10 and IL-1RA [145]. Moreover, a recent study revealed that, in response to excessive oxidative stress, neutrophils are shown to have high levels of SELENOR, while decreased levels might be associated with Alzheimer’s Disease (AD) [146]. In another study, also in response to an increased ROS action, SELENOR proved to be highly expressed in carcinoma cells, and, thus, it may enhance their survival. In addition, SELENOR expression upregulation enhances oncogenesis by stimulating proliferation via activating the MAPK pathway and metastasis by involving actin cytoskeleton dynamics [147,148]. In mice, SELENOR knockdown determines increased oxidative stress in the kidney and liver with severe hepatotoxicity in the liver [149,150].

In mice, SELENOR knockout led to the conditions of oxidative stress. Studies performed by Fomenko et al. showed that the liver and kidney are severely affected while the other organs’ redox status was not significantly changed [149].

### 3.5. Signaling Pathways Mediated by SELENOP

The organism has a so-called “selenium pool”, which is under liver control in order to provide the selenoproteins with selenium. In case of a selenium deficiency, the liver, as the central regulatory organ, releases SELENOP into the plasma. The distribution of SELENOP in the corresponding extrahepatic tissues is accomplished by two members of the low-density lipoprotein receptor family, apolipoprotein E receptor-2 (apoER2, LRP-8 or low-density lipoprotein receptor-related protein) and megalin (LRP-2, low-density lipoprotein receptor-related protein 2), that bind SELENOP forms and make their endocytosis easier [151,152].

Each of these two receptors has a different distribution in the organism, different SELENOP-binding properties, and plays a different specific role in the physiology of SELENOP.

Regarding megalin, studies have demonstrated its presence on the apical surface of the renal proximal convoluted tubule (PCT) cells, being responsible for the reabsorption of proteins and other ligands from the glomerular filtrate. In addition, megalin is expressed in the brain and other tissues. In the kidneys, via the megalin receptor, the NH_2_-terminal domains of SELENOP are filtered and reabsorbed in the proximal convoluted tubule (PCT) [81]. The experiments that were performed showed that the brain, under selenium deficiency conditions, is able to retain selenium better than any other tissues studied [153,154]. However, knockout of either SELENOP or apoER2 lowers brain selenium drastically, from about 120 ng/g to 50 ng/g, and mice become susceptible to severe neurodegeneration (when selenium decreases to 35 ng/g) and even cell death when they are fed with a low-selenium diet, also suggesting that apoER2-mediated endocytosis of SELENOP is essential for the retention of selenium by the brain. [155,156,157].

ApoER-2 is found in the brain neurons and at the blood–brain barrier [99,158].

Recent studies demonstrated that is mandatory for apoER2 to act in tandem with SELENOP, apoER2 being necessary to transport selenium across the blood–brain barrier, and allowing the SELENOP to concentrate selenium in brain neurons, thus managing to supply neurons with selenium and protect them from degeneration [99].

In the brain, SELENOP is also expressed by astrocytes and is transported through the blood–brain barrier by binding to LRP-2 (megalin), which is a multiligand receptor, or to apoER2 receptor (LRP-8) with a membrane receptor function [159].

ApoER2 is concentrated in the testis in Sertoli cells and has a very high expression in these cells [152]. Sertoli cells form a blood–testis barrier and uptake the nutrients from the systemic circulation to distribute them to the germ cells; the mechanism of the SELENOP uptake by Sertoli cells also involves apoER2-mediated endocytosis [152].

A so-called selenium “tissue hierarchy” mentions the brain at the top and the liver at the bottom, and is based on the ability of the tissues to uptake and retain selenium when confronted with selenium-deficient conditions [81]. The testis, which produce and release selenium-rich spermatozoa, have a very high expression of apoER2 mRNA. Large quantities of SELENOP are found also in the bone marrow because the blood cells contain selenoproteins, and in the placenta in order to provide selenium to the fetus [79]. Moderate amounts of selenium are found in the brain and muscles, whereas in the liver and kidneys, the quantities are minimal because these tissues acquire selenium mostly in other ways [81].

In mice, regarding the maternal–fetal transfer of selenium, the placenta picks up SELENOP from the maternal circulation via apoER2-mediated endocytosis [79]. The visceral yolk sac and thereafter the placenta take up both SELENOP and Gpx3 (glutathione peroxidase 3) from the uterine fluid in a way that is not dependent on apoER2 or megalin and appears to take place via bulk pinocytosis [79]. It is to be mentioned that it seems it is the only reported case in which Gpx3 acts as a selenium transport protein. Knockout of SELENOP at the dam level under an adequate selenium diet depresses fetal selenium [79].

## 4. Conclusions

Selenoproteins are not just simply antioxidant enzymes. Selenoproteins perform many functions that involve thiol-based redox signaling, removal of hydrogen peroxide, selenium transportation, monitoring of the process of reducing Cys residues in cytosolic and mitochondrial proteins, protein tridimensional folding, ER-associated protein degradation, repair of the damaged proteins, the correct formation of the disulfide bonds, hormone activation, and a lot of many functions. The essential micronutrient selenium has a vital role in human health and its role is accomplished by selenoproteins consisting of the selenoproteome and also by the combined actions of them. Selenoproteins have become potential targets for new therapies for different diseases caused by the dietary and general status of selenium, so selenium’s status is always of great importance. Future research is needed to better characterize and understand the complexity of the structure and function of selenoproteins for improving both human and animal health.
